# Nursing practicum equity for a changing nurse student demographic: a qualitative study

**DOI:** 10.1186/s12912-022-00816-2

**Published:** 2022-01-30

**Authors:** L. Andrew, J. Dare, K. Robinson, L. Costello

**Affiliations:** 1 Edith Cowan University School of Medical and Health Sciences, 270 Joondalup Drive, Joondalup, WA 6027 Australia; 2 Edith Cowan University School of Arts and Humanities, Joondalup, WA Australia

**Keywords:** Nursing, Student, Practicum, Family, Finances, Stress, Women, Australia, Nursing workforce, COVID-19

## Abstract

**Background:**

The nursing practicum (clinical practice) is an essential but often highly stressful aspect of the nursing degree. A review of the published literature reveals a strong focus on the stressors that originate within the practicum environment, rather than the student’s life outside the university and practice setting. This article reports on an Australian study, completed before the COVID-19 pandemic, of the university experiences of undergraduate women nurse students with family responsibilities. The findings reveal the importance of factors outside the university on the women students’ practicum experience and their ability to engage and achieve.

**Methods:**

The study was qualitative, guided by Gadamer’s hermeneutic philosophy. Twenty-nine women students with family responsibilities (partners and children) were interviewed at two stages of their degree journey. Over 50 h of data were thematically analysed.

**Findings:**

The themes ‘family pressure’ and ‘practicum poverty’ describe the impact of domestic work, family finances and practicum organisation on student stress, wellbeing, achievement, thoughts of attrition, and family tension. These findings are particularly pertinent to Australia and other developed nations where the nurse student demographic continues to age. An interpretation of these findings against the recent impact of COVID-19 on nurse education and women’s life choices reveals the likelihood that these difficulties have intensified for women students with family responsibilities since the pandemic began.

**Conclusions and Recommendations:**

Many developed nations, including Australia, are increasingly reliant on older women nurse students to maintain the future graduate nursing workforce. This change in nurse student demographic to the mature-age student requires a revision of the organisation of the nursing practicum. Recommendations to nurse education to improve practicum accessibility for women students who have family responsibilities include the application of a flexible and collaborative approach to practicum organisation and communication. Wider recommendations to Government include a revision of the way the nursing student is financially supported during the practicum. Further research that explores the practicum experience for women nurse students during and following the COVID-19 pandemic is also recommended.

## Introduction

This article presents the findings of an Australian study of undergraduate nurse students that reveal women with family responsibilities experience a range of stressful situations that impede their access to, and satisfaction with, the clinical practice component of their degree. The financial, family and university factors that influence the women’s capacity to engage in the practicum, and their experiences of stress are highlighted. These findings and their implications are considered against the backdrop of the recent impact of COVID-19 on the clinical practice placement (termed ‘practicum’), the change in nurse student demographics, and the imminent nursing workforce shortage. Recommendations are made to promote future practicum equity and with it, the retention of the growing cohort of mature-age women nurse students who begin university with family responsibilities.

## Background

The international shortage of nurses and other vital healthcare professionals is of profound concern and is projected to increase to an estimated global deficit of 12.9 million nurses, midwives and doctors by 2035 [1]. Countries such as Australia, which have an ageing nursing workforce, are expected to face particular nursing workforce shortages [2]. According to the Organisation for Economic Co-operation and Development, “increasing investment in nursing education is particularly important as the nursing workforce is ageing in many countries and the baby-boom generation of nurses approaches retirement” ([3], para. 1). In Australia, around 40% of nurses and midwives are aged 50 years or over [2, 3]. The associated surge in retirement of practicing nurses in recent years means the continuation of the graduate pipeline is crucial to maintain this workforce [2, 3, 4].

The nurse student demographic has also continued to age as school leavers have turned away from nursing as a potential future career [5]. A review of the international nursing literature has revealed young adults aged 15 to 24 years old across developed nations tend to hold a dated and incorrect perception of nurses as low status and less intellectually able that other health professions [6]. Although the age of nurse students is not centrally or routinely recorded in Australia, a report of 2014 university data demonstrated three times more non-school leavers than school-leavers had applied for an undergraduate nursing degree [7]. This demographic shift suggests an unprecedented number of students now begin a nursing degree while living with partners (and children), rather than with parents.

Studies from Hong Kong [8] and the USA [9] reveal that nursing students of all ages report higher levels of anxiety than students in any other discipline. The international nursing literature has identified the practicum as the most stressful aspect of the degree [10], with this stress detrimental to student health, wellbeing and performance, and a trigger for attrition [11, 12].

The international research on practicum stress tends to focus on the quality of the experience itself, such as the student-supervisor relationship, whether the environment was welcoming or not welcoming nature, and the assessment of practice [^13^, ^14^, ^15^]. However, research into the university experiences of mature-age women students suggests a more complex and interconnected range of factors that contribute to practicum stress for this student group. The practicum represents a period where the student is expected to commit to full-time engagement for weeks to months at a time and may be a particularly stressful period of the nursing degree for women with family responsibilities. A lack of male partner support with domestic responsibilities (housework and childcare) can restrict the woman’s capacity to meet the time demands of their degree, especially during the practicum [16, 17, 18]. This lack of support can cause conflict within the intimate relationship, which can further heighten the woman’s perceived stress.

A further issue that may disproportionately affect the practicum experience for women with family responsibilities is its financial implications. The practicum in Australia is unpaid and is organised as a full-time commitment, thereby restricting students’ ability to undertake paid work. Australian data from 2005 to 2018 (inclusive) reveal over 70% of domestic undergraduate students were engaged in full time paid work [19], with a study of undergraduate nurse students reporting the primary reason behind the decision to engage in paid work during the degree as financial, with students citing mortgage repayments and family support as major driving factors [20]. A further study, this time with 160 nurse students at a single Australian university, reported “the majority of students struggle(d) financially during clinical placements” ([21] p.1). It is likely that students with family responsibilities will be more affected financially than single students, especially those single students who live in their parents’ home.

This article reports on the practicum experience of women nurse students with family responsibility in an Australian School of Nursing and discusses their implications against the ageing nurse student demographic and the graduate nurse workforce shortage. Findings are also considered against the backdrop of the COVID-19 pandemic and its ongoing impact on nursing stress and practicum availability.

## Method

The findings reported in this article come from a larger qualitative Australian study completed in 2016, that aimed to understand the university experiences of women nurse students who began university in a heterosexual intimate relationship [16, 18]. This larger study aimed to understand gender relationships between cis women and men and the influence of these relationships on cis women student university experiences. The study methodology was guided by Gadamer’s hermeneutic philosophy, which supports the development of authentic and meaningful understanding of experiences from the participant’s perspective [22].

### Data collection and analysis

Ethics approval was obtained from the University's Research Ethics Board. The lead author, who interviewed all participants, was not employed in the School of Nursing during the study and therefore there was no power relationship influencing participants’ decisions to take part.

Participants were women nurse students of a Bachelor of Science (Nursing) degree at a Western Australian university, who began their degree in a heterosexual relationship. A purposive sample of 29 women were interviewed in their second year of study, with 23 of these women completing a second interview in their final semester, the other six declining due to competing time commitments. All participants in this study completed their degree. Interviews involved in-depth, face to face conversations that explored the university experiences from commencement to the final semester of study. Interviews were audio-recorded and transcribed verbatim. Interviewing and data analysis occurred concurrently. Participant recruitment ceased once data saturation was achieved, This was decided as the point where no new themes or codes could be developed from the data of four consecutive interviews and interview summaries were shared with participants to ensure accuracy. In total, over 50 h of data were organised using the NVivo software. This data was coded and thematically analysed by the lead author.

## Findings

### Participant demographics

The average participant age was 34 years, with an age range of 19 to 48 years. All lived with a male partner at the start of the degree; the average length of their relationship at commencement was nine years. Twenty-two women had dependent children living in the family home. Ten were migrants and nine had partners in fly-in-fly-out (FIFO) work. Eighteen participants studied full-time, 22 were in paid work. Participants worked on average 23 h per week. Four women worked over 38 h a week, and three of these four also studied full time.

### Themes

The two overarching themes, ‘family pressures’ and ‘practicum poverty’, explore the impact of the family responsibilities and family finances on the accessibility of the practicum, student achievement, thoughts of attrition, stress and wellbeing and family tensions. The impact of the organisation of the practicum by the university and healthcare providers is also explored across these themes. Where direct quotes are used to illustrate themes, participants are described as follows: (pseudonym, age, work (full time FTW or part time PTW), dependent children at home (CH) and FIFO partner (work, CH and FIFO only included where applicable).

#### Family pressures

The first theme describes how the time demands associated with the women’s family responsibilities compounded issues related to the organisation of the practicum, influenced practicum accessibility, student stress and individual and family wellbeing, as well as leading some participants to contemplate withdrawing from the course.

The women in this study described how they continued to take responsibility for the domestic work and childcare once they began university [see 16, 18]. Participants reported that in their relationship, their male partner did not regard themselves as responsible for domestic duties, and instead viewed their role as largely restricted to the main income generator:

My husband was very much the breadwinner, bringing in the money, and I was the housewife (Chantelle, 38, PTW, CH3, FIFO).

Participants explained that they had to juggle family, university and work demands on their own, and their capacity to manage these competing demands was further exacerbated by the way practicums were organised and communicated to students. For example, the university did not provide students with the opportunity to request which rotation (part of the semester) they would undertake the practicum, and typically communicated this information at the start of each semester, immediately before the practicum commenced. This left participants with little time to make arrangements for managing paid work and childcare. Late communication of shift details from the hospital or other practicum provider was also commonplace, as indicated by this quotation:

You don’t get your roster [of shifts] until a week and a half before you go. I mean some hospitals; you get it on the day you turn up there (Candice, 40, PTW, CH3, FIFO).

Participants explained how the timing of shifts was a particular issue for participants with young children. As early shifts began at 7.00am and childcare rarely opened before 6.30am, this left little time to travel to the practicum placement each morning. The university’s allocation of practicum during school holidays also meant finding a childcare provider with little notice.

Some participants experienced periods when they had no practical support from their partners during their practicum. This was particularly the case for nine participants whose partners worked in ‘fly-in-fly-out (FIFO) positions. FIFO describes employment that, because of distance from home, requires the worker to remain at the work site for weeks or months at a time. Partners would often be absent from home at remote worksites, often for weeks at a time. In common with non-FIFO partners, these men also tended to offer little support when at home. A compounding issue for participants in these relationships was the tendency for FIFO partners to want to spend large amounts of time with them when they were home, which limited the time available for study. Kim (40, CH2, PTW, FIFO) for example, described how her partner was very demanding of her attention when at home and resented the time she devoted to her degree. The tension this created within the relationship left her “working like a madwoman” during the times he was away with work.

A similar situation was experienced by participants who had migrated to WA, and who had limited local extended family. These participants were accustomed to coping without extended family support. Rebecca, a migrant from the UK exclaimed:

We do tend to manage things on our own, ‘cause we’ve nearly always done that (Rebecca, 43, PTW, CH3)

Physical distances to practicum were a further source of stress. Participants reported that the university did not consider students’ home addresses when allocating placements, with participants often having to travel long distances. Brenda (46, CH2, FIFO) recalled students’ panicked reactions when practicum details were released:

When the prac. placements go up you get a flood of nurses saying ‘Oh God, I got south of the river and I live north can anyone swap please? I can’t get there’. How they expect loads of us to travel north who live south and then another load who live south to travel north it doesn’t make sense. I mean I know they have lots to organise, lots of placements, but that doesn’t make sense.

The students described how the difficulties associated with their practicum location were further compounded as many of the major hospitals did not allow student parking, and participants were therefore restricted from using their own car. Participants explained that the additional time needed for using public transport further reduced the time available to study and to spend with family, leaving many feeling exhausted. Jilly (45, PTW, CH 2) for example, explained:

So, I was down at [hospital] and you’re not allowed to park, so that’s a bit of a nightmare, because it’s like up at 4.30 to drive to the train then a bus to start at 7am. Then home again, you’re not getting home till 5pm with the transport. So, it’s a really long day. You just find when you’re on those shifts, you’re neither use nor ornament when you come home. By the time you come home, get yourself sorted for the next day, you’re in your bed at 8pm, so it’s ‘hi, bye’

Students completed theory units and assessments at the same time. This concurrent running of theory and practicum units further reduced participants’ ‘down time’:

You’re out on prac doing early, late [shifts] then to come home and stay up all hours of the night and try to do uni stuff. (Jilly, 45, PTW, CH 2)

Participants recounted how, as the length of the practicum increased throughout the degree, the stress associated with managing the combined expectations of university and home life took its toll on their health and wellbeing. Participants commonly talked about sacrificing sleep and social time with family and friends and resorting to convenience foods. Complaints of subsequent weight gain, social isolation and exhaustion were common. These combined and cumulative stressors toll their toll on some participants’ mental health. Paige (24, FTW, FIFO) for example described how she “ran into a bit of trouble” as her coping mechanisms were worn down in the later stages of the degree, forcing her to reduce her study load to part-time, while Candice (40, PTW, CH3, FIFO) recalled friends who found these competing demands impossible as the practicum lengthened:

Some really good people who pulled out of their degree in later semesters because they could not organise time with family commitments.

These comments reveal how conflict with partners and children arose because of the time the women’s study and practicum commitments took away from family, coupled with the lack of partner domestic support. Towards the end of their degree, eight of the participants’ intimate relationships broke down [discussed in detail in 15] requests for assistance from university staff during the practicum resulted in mixed responses. On separating from her partner, Frankie requested more practicum late shifts, to fit in with childcare opening hours. The first staff member she approached refused to help, explaining that nursing was not a flexible degree. This rigid approach left Frankie in a situation where her only option was to leave university – eventually remedied when her mother offered her a temporary home. Fortunately for Frankie, in the following semester, a different staff member was more accommodating and worked with her to rearrange the location and times of her practicum.

#### Practicum poverty

Around half the participants, especially those with young dependent children, and whose partners worked in low-paid occupations, indicated that they could not cease paid work or drastically reduce their hours to help them better manage during the practicum. These participants reported using a range of strategies to enable them to complete their practicums.

A common practice of accepting additional paid work before and after practicum weeks helped compensate for the loss of income during the practicum for some participants. A downside of this strategy, however, was that it left even less time available for study; which participants reported led to lower grades than usual in academic assessments. Kylie (20, FTW, FIFO) for example, worked 30 h a week to meet the family mortgage. In the weeks preceding and following the practicum she increased her work to 40 h per week. As she explained, during these periods study became impossible, because she “simply didn’t have time for uni”. While Kylie explained this had a detrimental impact on her grades, she explained that increasing her work hours had been unavoidable.

In the first year of the degree, some participants were able to use their annual leave to complete the comparatively short early practicums. In the second and third year however, practicums ran for a total of eight weeks and 10 weeks respectively, making fixed employment contracts difficult to maintain for these participants. One solution was to move to casual contracts, as described by Marla (21, PTW, FIFO):

The (casual) job that I have now is more supportive and that’s also why I went for it, because they are a lot more supportive of uni… Whereas the last work I was at, they wanted me to do certain days, and of course if you’re timetable changing every semester it was hard. They weren’t too happy about me going on prac.

However, casual work brought additional problems such as the loss of important employment benefits such as sick pay and annual leave entitlement, which added to financial difficulties and personal fatigue.

Participants who stayed in their original work contracts resorted to other ways to accommodate practicums. This included using unpaid leave entitlements; a solution, however, that caused periods of financial stress to the family. For example, Ros (40, PTW, CH 2, FIF0) combined her study leave, long service leave and annual leave entitlements to attend the final year practicum. While this strategy enabled her to complete the practicum component of the course, the resultant loss of free time in this final year left her feeling stressed and exhausted.

Participants’ reduced income following relationship breakdown exacerbated financial stress. Some of the eight women who separated from partners increased their paid work hours to support themselves and their children. While this alleviated some financial stress, it also created a further barrier to practicum accessibility. Other participants were able to move in temporarily to their parents’ home, and while this home brought financial relief, it also meant that some had to travel much further to attend practicums.

## Discussion

This study has highlighted the financial and family difficulties that women nursing students with family responsibilities experienced during their practicum. Study findings also reveal how issues external to the university inter-relate with the structure of the practicum to impact on student stress, wellbeing, academic achievement, financial stress and family tension, as well as potentially contributing to attrition. These findings are now interpreted against the backdrop of the changing dynamics of the nursing workforce and the impact of the COVID-19 pandemic on these women’s lives and on nurse education.

In 2011 an Australian action research study by Kenny et al. [23] reported the top reasons for attrition among mature-age nurse students were financial difficulties (25.6%), time constraints (23%) and lack of family support (10.3%). The present study reveals the practicum is a period of the nursing degree when these issues are particularly intense for women with family commitments. Nursing studies appear to have largely overlooked external issues as causes of stress, focusing more on factors within the clinical environment, such as unhelpful or unsupportive staff colleagues or supervisors (see [24, 25]). With most nurse students now mature-age, the study’s findings indicate a shift is required in the focus of research into the practicum experience to factors, that originate outside the university and practicum, as well as the compounding impact of the practicum organisation by the university and practicum healthcare provider.

On commencing their nursing degree, the women in this study took on an increased burden of tasks. The current COVID-19 pandemic has highlighted and exacerbated the impact of the ongoing unequal division in domestic duties in Australian society on women’s lifestyles and life choices [26]. The closure of schools and childcare services during the COVID-19 pandemic has increased the time demands placed on women with children [26]. It is likely that the increased domestic load experienced by women as a result of the pandemic will exacerbate experiences of practicum stress.

Despite previous calls for timely and explicit communication of practicum information with mature-age nurse students [23, 27, 28] study did not provide evidence of effective communication of practicum-related information. Withholding or delaying information about practicum times and locations, denying student participation in the planning of their own practicum rotation dates, and refusing parking access at hospitals, created structural barriers to participants’ successful completion of their practicum, caused significant stress, and effectively deprived these women of the opportunity for self-determination in their student and personal lives.

The importance of parental support to women nurse student degree progression has been described in previous research [23, 27]. Although crucial to some of the women in the present study, other participants did not have parents who lived locally and therefore could not access this form of support. In addition, the impact of partner FIFO work on partner support indicates the need for local understanding of the student situation in curricular planning.

The study also reveals the extent to which the women’s academic achievements, wellbeing and family relationships were affected during the practicum. Although all participants in this study completed their course, many reported considering withdrawing themselves, and knew of mature-age women student peers who had withdrawn because of practicum-related stress.

Previous research with mature-age nurse students has highlighted the importance of greater flexibility in degree delivery, such as part-time practicum offerings, and school hour timetabling [23]. This flexibility, however, depends on practicum availability. With the hospital system already stretched, the COVID-19 pandemic has placed added pressure on practicum availability to the extent that the degree’s mandatory practice hours have been adjusted [29]. In Australia, this has meant a reduction in the minimum clinical practice hours required for graduation, from 800 to 776 [30]. For the foreseeable future, the lack of practicum availability will reduce accessibility and choice. Students may find they have to accept a placement further from home, with details changing at the last minute as availability alters. This will particularly affect students with limited capacity available to travel long distances, those who rely on regular income and those who have childcare considerations.

While crucial to degree continuation, the strategies employed by participants to support their income had negative consequences. Rochford et al. (2009) [31] have reported an inverse relationship between the number of hours in paid work per week and nurse student academic achievement. In the present study, students who increased their paid work hours before and after the practicum to ensure sufficient family income, similarly reported a drop in grades. Other strategies, such as relinquishing permanent positions in favour of casual work that fitted around the dynamic and sometimes unpredictable requirements of the practicum enabled continuation, but left students in a vulnerable situation financially, with no guarantee of regular work. Compounding this, the loss of paid sick leave and annual leave left some participants exhausted. In Australia, women are more likely to work in insecure casual positions. The COVID-19 pandemic has heightened the susceptibility of casual workers to being laid-off at short notice without the legal protection or financial security offered to people with permanent employment contracts [26]. These findings demonstrate how women nurse students may currently be exceptionally vulnerable to practicum financial stress.

Any loss from the pipeline supplying the graduate nursing workforce through student attrition will add to the pressure on health systems and the needs of its population [32]. The World Health Organization [2] has called on governments to provide financial levellers to increase enrolments among students who rely on an income during their time at university. At first glance, recent changes to Australian study fees may go some way to achieve this. From 2021, nursing fees have been reduced by 42% to $3950 per year [33]. While this may make nursing more attractive to new students, it does not address the immediate day-to-day financial difficulties associated with the practicum. An increase in enrolments may therefore be matched by an increase in attrition. A more sustainable and effective solution could be a means-tested funded practicum, or the option of part-time practicum offerings.

The surge in nurse student applicants reported by Australian universities during the COVID-19 pandemic is also promising [34], but is tempered by two facts; firstly, any increase in enrolments will put more pressure on already limited practicum availably, and secondly, the increase in enrolments is likely to be greater in older applicants. Although an age breakdown of applicants is not available in Australia, a similar increase in UK nursing degree applicants indicates that between 2020 to 2021, the greatest increase in nurse student applicants was in older women (39% increase aged 35 and over, and 45.3% rise aged 25–29-year-old, compared to 26.7% in 18-year-old applicants) [35]. To ensure an equitable university experience for these students, the organisation of the practicum needs to consider the impact of their responsibilities outside the university on their wellbeing, achievement and attrition.

Lastly, the study’s findings highlight the impact of practicum stress on student mental health. The bidirectional relationship between student health (with an emphasis on mental health) and stress during the practicum has been previously highlighted in a Taiwanese study carried out pre-COVID-19 [10]. Here, high levels of practicum stress was closely correlated with low health status. This Taiwanese study, however, was carried out with young students (mean age of 19 years), who were unlikely to have family responsibilities that can compound the situations of stress within the practicum environment.

Emerging international research has revealed that during the COVID-19 pandemic, there has been an increase in nurse student anxiety, prompted by uncertainty, changing situations and expectations, increased mortality, fears of personal safety and disruption to the practicum [36]. The consequences of the pandemic, including employment insecurity and loss of casual work, home schooling and reduced practicum choice and availability may exacerbate the family and financial difficulties experienced during the practicum by women nurse students with family responsibilities. These findings reinforce the immediacy of the requirement of nurse education to reconsider the requirements of its growing cohort of older women students and the impact of practicum organisation and communication on their wellbeing. It is also important to note here that this study was limited to an exploration of heterosexual student experiences. The authors acknowledge the importance of research into the unique experiences of LGBTQI + nursing students.

## Conclusions

The study has identified how the practicum represents a highly stressful aspect of the nursing degree for women students with family responsibilities. Family and financial factors originating outside the university are compounded by poorly communicated and unpredictable practicum organisation, to induce a stressful situation of reduced practicum accessibility, increased financial insecurity, family tension and threats to wellbeing and academic attainment, and may increase the risk of attrition. The COVID-19 pandemic has highlighted and, in some ways, amplified these issues, and will continue to do so for the foreseeable future. For countries with an ageing nurse student demographic, there is an immediate need for nurse education and healthcare providers to acknowledge and mitigate the university factors that create this stress and disadvantage these students. Such actions may mitigate future nurse workforce shortages and address issues of student inequity within higher education.

## Recommendations

Recommendations to redress the inequity facing women with family responsibilities during the practicum include:

- Communicate practicum details in a timely manner and avoid the concurrent delivery of theory units and assessments
- Offer a consistent flexible approach with students experiencing family and financial difficulties
- Listen to and work with students who have competing and unavoidable responsibilities outside university, to identify accessible practicum times and locations
- Research student stress, mental health and attrition associated with the practicum in the current and post COVID-19 pandemic environment.
- Consider a means-testing approach to supporting the financial needs of nurse students during the practicum

## Data Availability

Data are in the form of 52 participant interviews. These are confidential and not accessible to the reader. Requests to access the data should be made in writing to lead author LA.

## Abbreviations

FTW: Full-time work
PTW: Part-time work
CH: Dependent children of research participants
FIFO: Fly-in-fly-out work
